# Converging on child mental health – toward shared global action for child development

**DOI:** 10.1017/gmh.2017.13

**Published:** 2017-10-19

**Authors:** G. Belkin, L. Wissow, C. Lund, L. Aber, Z. Bhutta, M. Black, C. Kieling, S. McGregor, A. Rahman, C. Servili, S. Walker, H. Yoshikawa

**Affiliations:** 1 Division of Mental Hygiene, New York City Department of Health and Mental Hygiene, New York, New York, USA; 2 Center for Mental Health Services in Pediatric Primary Care, John Hopkins Bloomberg School of Public Health, Baltimore, Maryland, USA; 3 Alan J Flisher Centre for Public Mental Health, University of Capetown, Capetown, South Africa; 4 Steinhardt School of Culture, Education, and Human Development, New York University, New York, New York, USA; 5 Global Child Health Centre, Toronto's Hospital for Sick Children, Toronto, Canada; 6 Department of Pediatrics, University of Maryland School of Medicine, Baltimore, Maryland, USA; 7 Graduate Program in Medical Sciences, Universidade Federal de Ciencias da Saude de Porto Alegre, Porto Alegre, RS, Brazil; 8 Institute of Child Health, University College London, London, UK; 9 Child Psychiatry, University of Liverpool, Liverpool, UK; 10 Mental Health and Substance Use, World Health Organization, Geneva, GE, Switzerland; 11 Tropical Medicine Research Institute, University of the West Indies, Mona, Jamaica

**Keywords:** Public Health approach, systems-thinking, Mental Health, Child Mental Health, Prevention, Promotion, Social Emotional Development, Child Development, Social Determinants of health, Global Action, Governance, Task-shifting, Quality Improvement

## Abstract

We are a group of researchers and clinicians with collective experience in child survival, nutrition, cognitive and social development, and treatment of common mental conditions. We join together to welcome an expanded definition of child development to guide global approaches to child health and overall social development. We call for resolve to integrate maternal and child mental health with child health, nutrition, and development services and policies, and see this as fundamental to the health and sustainable development of societies. We suggest specific steps toward achieving this objective, with associated global organizational and resource commitments. In particular, we call for a Global Planning Summit to establish a much needed Global Alliance for Child Development and Mental Health in all Policies.

## Rationale for an emerging global child mental health agenda

Reducing high levels of preventable child deaths remains a crucial part of global health efforts, especially in low- and middle-income countries (LMICs). As mortality has fallen, the global child health agenda has turned toward healthy social and emotional, as well as physical, development. This shift in focus should be an opportunity to take long-neglected and unmet child and adolescent mental health needs, more seriously. The 2015–2030 Sustainable Development Goals (SDGs), have taken a step in this direction in calls to ‘promote mental health and well-being’ in target 3.4 and ‘strengthen the prevention and treatment of substance abuse’ in target 3.5 (UN, 2015).

Mental health is not only an absence of disorders, but also the possession of beneficial attributes, including the capacity to form positive and meaningful relationships, develop effective emotional, behavioral, and attention, and maintain a sense of self-esteem and self-efficacy. Thus, besides treating disorders, mental health interventions should support the emotional and cognitive repertoire that enables children to become adults who can adaptively respond to stress and be productive. Mental health measures, interventions, and providers are therefore positioned to be an important bridge between investments in child development and broader health and social development success.

## Simultaneous epidemiologic and social transitions

The broadening of attention within the global child health field beyond child survival reflects great progress: a 40% reduction in infant mortality and an almost 50% reduction in under-5 mortality in the last decade (Kuruvilla *et al*. 2016). As with shifting burdens of disease and mortality in adults, these achievements have uncovered other public health priorities among children. Improved survival increasingly means that governments, especially those of lower resourced countries, pivot from mortality to morbidity, from surviving to thriving. The public health importance of successful early healthy emotional development and resilience throughout childhood will only increase in these settings. Even with still underdeveloped data gathering in most countries, it appears that suicide is among the leading causes of mortality in adolescence for girls and boys, especially at ages 15–19 (World Health Organizaton, 2014); depression and anxiety are among the top five causes of years lost to disability in 10–14 years old, and depression is among the top five global causes of disability-adjusted life years for adolescents (Dick & Ferguson, 2015).

The emergence of mental disorders is not just a function of their unmasking by increased survival but also of increasing challenges to healthy social and emotional development. A recent review of national data in the USA identified a ‘new epidemiology of childhood’, where ‘adverse social, economic, and child-rearing conditions … are loading children down with preventable illness, disability and dysfunction’ (Halfon *et al*. 2014). Children and youth in LMICs face an even more daunting environment shaped by increasing economic inequality, stagnating or reduced opportunities to escape intergenerational poverty, sexual abuse and exploitation, premature workforce enrolment and trafficking, and underfunded and underdeveloped educational systems (WHO, 2015). Globalization promotes rapidly changing social and gender norms, and political instability strains and separates families and communities. In too many regions, childhood is marked by forced and prolonged displacement and migration, most often under conditions of sustained threat and deprivation, with UNICEF recently estimating 50 million children ‘uprooted’ globally – over half due to violent conflict (UNICEF, 2016).

All these forces will only accelerate the degree to which mental health is a key contributor to health for children.

## The brain as the link between the environment and health

A growing evidence base supports mental health as a bridge uniting child development goals (McEwen, 2012). The social determinants of *health* – both positive and negative – operate through mechanisms of *emotions and behavior*, through modulation of resilience and stress responses. Conceptualising mental health as the link between social conditions and health outcomes provides new opportunities to redesign and integrate mental health treatment and prevention/promotion capabilities within platforms and policies for child health and development. Such integration is essential to optimize the bridging value of mental health.

The case we will therefore make in the lead-up to a call for bold global action is that:

1. Mental health promotion is inextricably linked to broader child development goals, including survival and successful transition to meaningful, productive adulthood. Healthy societies depend on mentally healthy populations.
2. The science guiding child mental health has a lifespan perspective that begins prenatally by ensuring maternal mental health alongside of physical health and safe deliveries. It goes on to promote children's physical, cognitive, and psychosocial health by promoting consistent and responsive caregiving. It identifies and addresses the social and structural determinants that threaten the nurturing children receive from their families and communities, and it identifies and promptly treats mental disorders should they emerge.
3. These promotion, prevention, and treatment activities can and should purposefully converge with other child development funding, policy, and delivery in a comprehensive global strategy that focuses on three key areas: *integration*, *improvement*, and pooled *investment*.

### Mental health, physical health, and child development

In 2003, the Bellagio Child Survival Group opened a *Lancet* series calling for sustaining efforts in child survival in the face of competing investments in HIV, TB, and malaria. A subsequent *Lancet* paper, in 2007, from the then newly formed Global Child Development Group, warned that more than six million annual preventable child deaths highlighted in the 2003 paper were only ‘the tip of the iceberg’. The authors estimated that annually more than 200 million children under 5 years survive but fail to reach their developmental potential (Grantham-McGregor *et al*. 2007). In 2016, the figures were revised upwards to 250 million, and although progress in early childhood development research, programs, and policies had been achieved in the last decade, services for young children are inadequate and inequitably distributed (Black *et al*. 2017).

The Global Group's estimates initially focused on what they called ‘cognitive development’. While that originally referenced primarily IQ and school performance and readiness, subsequent Group reports successively referenced further attention to socio-emotional health by identifying support for maternal mood, child attachment, and child emotional resilience as priority, actionable areas to promote optimal cognitive developmental potential (Walker *et al*. 2007; Engle *et al*. 2011).

These traditionally separate strands of cognitive and emotional health are increasingly understood as comparably important also inextricably linked core aspects of human capital. For example, economists such as Flavio Cunha, James Heckman, and their colleagues (Cunha & Heckman, 2010) argue that maximizing an individual's social, intellectual, and economic potential depends not only on skills in processing and manipulating information (acquired in well-functioning schools), but also on the regulation of emotions, behaviors, and relationships (Dercon & Lives, 2011). Successful acquisition of both of these sets of capabilities is closely related. The emotional and relational skills enhance the acquisition of processing skills, and are independently associated with later life success (Heckman *et al*. 2006).

The field of developmental psychopathology has itself similarly advanced over the past 25 years, gaining understanding of how child mental health problems develop, including specificity of environmental risk factors amenable to prevention and the interplay of biological, genetic, and contextual processes (O'Connell *et al*. 2009; Cicchetti & Rogosch, 2012). Mental health problems during childhood can disrupt developmental trajectories. Depression, for example, is characterized by poor self-esteem, a diminished sense of self-efficacy, and in many cultures, social isolation or rejection. Depressed children often experience academic problems that exacerbate their sense of failure and diminish access to social opportunity (Bethell *et al*. 2014).

Physical health is also integrally related to cognitive development and positive mental health. For example, infectious diseases, poor obstetric practices, and malnutrition all affect child mental health outcomes (Kieling *et al*. 2011; Walker *et al*. 2011). Inversely, healthy cognitive, social, and emotional development provides protection from somatic health problems through their influence on caregiving, nurturing, avoidance of individual risk behaviors, adherence to treatment, and the ability to invoke brain mechanisms that buffer exposure to acute and chronic stress (Repetti *et al*. 2002).

### Science behind prevention, treatment, and promotion

A growing line of research points to opportunities to act on these connections. There are periods of childhood during which exposure to adversities, or nurturing and stimulation, can especially impair or enhance development of both socio-emotional and information processing skills. Information processing may be more sensitive to exposures during infancy and the pre-school years, while socio-emotional skills may be more malleable into adolescence or even young adulthood (Del Giudice *et al*. 2011). That said, the existence of these periods does not mean that later interventions are ineffective, but they may have diminished or selective impact (Levin *et al*. 2014; Wachs *et al*. 2014). For example, attachment in the first years of life is a key driver of later socio-emotional health that can be improved through readily implemented methods of coaching parents (Kitzman *et al*. 2010; Lindsay *et al*. 2011). Programs that support parenting practices that promote attachment and support child socio-emotional development have a positive impact on children and their parents throughout both school age and adolescence (Whittle *et al*. 2014).

Poverty is one of the most pervasive risks for poor developmental outcomes, and it has an impact throughout childhood. Poverty, still too often itself driven by structural discrimination and racism, exerts its effects at many levels, including reduced parental stimulation, increased parental and child stress, poor nutrition, increased exposure to somatic health risks, and decreased educational opportunities (Walker *et al.* 2007; Dercon & Krishnan, 2009). Children living in poverty are often cut-off from community and social resources through residence in isolated rural and urban areas and lack of transportation.

In parallel with efforts to reduce poverty and exclusion (e.g. employment, housing and income security, universal health access), there are a number of possible interventions to moderate the impact of poverty on child mental health. These include increasing the availability of pre-natal care and attended births; early food supplementation; helping parents provide enhanced verbal, social, and cognitive stimulation and other forms of parenting support or training; promotion of both child and parent attachment; emotion-handling and problem-solving skills, provision of or enhancement of early childhood/pre-school education (Barry *et al*. 2013; Fazel *et al*. 2014).

This broad menu of interventions rests on a narrower set of overlapping skills that are accessible to a potentially diverse set of workers and providers, allowing the possibility for broad ownership of a comprehensive strategy across a variety of workers and settings, such as schools, child care, nutrition supports, and other community services and networks. The synergies in cognitive and socio-emotional health that are key to successful child development can therefore be mirrored in their operational integration. For example, in high- and low-resourced settings alike, a body of proven skill packages and interventions that can be managed by school staff represent an enormous opportunity to scale proven methods of mental health promotion. These of course add to the protective effect educational achievement itself provides across these development domains.

In addition to promotion of child mental *health*, strategies for the expanded prevention and treatment of specific mental *illness* is also amenable to integration within other child health and development approaches and workforce. There is increasing evidence for primary and secondary prevention of mental disorders among children, especially anxiety and depression in children and adolescents. While this evidence is predominantly drawn from high-income country settings (Durlak & Wells, 1997; Merry *et al*. 2004; Cuijpers *et al*. 2005, 2008; Bayer *et al*. 2009, 2011), there is growing evidence showing effectiveness of similar mental health interventions for school-age children and adolescents in LMICs as well (Bolton *et al*. 2007; Tol *et al*. 2008; Catani *et al*. 2009; Jordans *et al*. 2010).

In particular, it may be possible to reduce the intergenerational transmission of depression and anxiety both through interventions with as-yet unaffected children and through treatment and prevention of depression and anxiety among parents, using integrated delivery models (Muñoz *et al*. 2012). Work by Rahman and colleagues have shown that lay community maternal health workers can deliver effective interventions for perinatal depression, exemplifying a scalable delivery opportunity that can carry and integrate several child development goals converging on mental health (Rahman *et al*. 2008).

Interventions in early childhood, middle childhood, and adolescence can also reduce the incidence of externalizing disorders, such as conduct and antisocial personality disorders. These can be implemented in a variety of settings (schools, clinics) and target both the individual and family (Yoshikawa, 1994; MacKenzie *et al*. 2002; Conduct Problems Prevention Research Group, 2011; Baker-Henningham *et al*. 2012). Whether through universal or selective prevention approaches, success in shortening the duration of mental disorder during adolescence contributes to reducing morbidity in adulthood (Patton *et al*. 2014), and cognitive development-focused interventions also show mental health impact in LMIC settings (Baker-Henningham, 2013).

Convergence of child mental health promotion, prevention, and treatment with overall survival and development strategies, is operationally and functionally compelling and feasible (Britto *et al*. 2017). It will increase the likelihood that the reciprocal connections across these domains of health are made for children worldwide.

### The strategy: integration, improvement, and investment

We suggest three areas for action to accelerate this convergence and usher in a global ‘new normal’ that operationalizes the bridging potential of a broad goal of mental health for all children. These three areas are: integration, improvement, and investment (see table 1 for summation). These areas capture lessons learned in scaling and spreading other global health priorities.

**Table 1.** Converging on mental health through integration, improvement, and investment

Integration through policy
− Adoption of ‘mental health in all policies’ approaches
− Leverage Sustainable Development Goals that support child/youth mental health, including:
○ Goals that promote child security and social–emotional development
○ Goals that promote child cognitive development
○ Goals that promote health care access
Integration through governance
− Incentives for intersectorial consultation and joint action, including opportunities for embedding mental health specialists in related agencies
− Shared evaluation plans building mental health measures into assessments of projects in related sectors
− Cross-sector program planning (e.g. across maternal and child health, adult and child mental health, housing and employment support, education)
Integration through delivery
− Use of task-sharing methods to increase skill range of cross-sector front-line staff
− Re-engineer mental health treatment and promotion interventions for use in non-specialist and non-medical settings
− Development and use of broad-based client assessment tools
− Further adaptation for children and youth populations of evidence-supported integrated behavioral health models in primary care (e.g. ‘collaborative care’)
Improvement
− Development of numerical targets for child mental health and wellness – UNICEF ECDI, WHO Global Mental Health Action Plan
− Embed indicators into national data systems or other shared methods for reporting
− Cross-sector and cross-national collaboration to identify potential new or best practices
− Adoption and spread to common use of ‘learning collaborative’ and other quality improvement and improvement science methods for these goals
Investment
− Establish a ‘Global Fund’ for parent and child mental health, and convene a preparatory Planning Summit for Child Development and Mental Health
− Continue to build the evidence base for the impact of mental health and related child social and cognitive development on short- and long-term economic and social development

#### Integration

Integration means more than collaborating or coordinating, but *unifying* investments and methods along common goals through shared information management, care operations, and decision-making (Britto *et al*. 2017). Improving child development could benefit from more opportunities to integrate mental health with overall child development aims in this way at the levels of *policy*, *governance*, and *delivery*.

In terms of *policy*, child mental health needs to be integrated with maternal health, child survival, health, nutrition, pre- and primary school, social development, and family-focused policies and solutions. Integration of this kind at a policy level requires a high degree of intersectoral ownership. This means not just commitment, but new, blended structures of governance and new cultures and capabilities of sharing of data, evidence, and measurable goals, across sectors. Aligning accountability through shared benchmarks and targets are central to facilitate this integration of mental health into policy. The SDGs invite such targets. Measurable improvement in social–emotional development for all children, for example, if applied across sectors as an accountable and measurable shared aim, can bend investment and institutional attention to the kinds of integration we advocate here.

This will also require integration not only in policies, but in methods and practices of *governance*. The visibility of the ‘health in all policies’ approach globally has yielded tangible effects, and methods, in how policy is formulated outside of the health sector. That approach has successfully positioned public health goals and methods to join disparate sectors around shared objectives. The adoption of a ‘Mental Health in all Policies’ approach (MHiAP) can have similar effects, and some governments, including the EU, have started to adopt or explore such an approach (Mantoura, 2014; McCray *et al*. 2015; Well-being, 2015). The international community should endorse adoption of a MHiAP approach as part of realizing the bridging power of child mental health resources and perspectives to child development.

Exemplars of this approach are growing: a national effort across municipalities in Sweden to integrate child and adolescent well-being into overall social policy (SKL); a cross-agency ministerial mental health council for the Provincial Government of Ontario (Minds, 2011); inclusion of mental health by India and South Africa in their adolescent and youth health strategies (MOHLTC, 2011), and the establishment of the New York City Mental Health Council, which brings together several dozen city agencies that cover areas ranging from policing and child welfare, to education, housing, and nutrition (McCray *et al*. 2015). These reflect scaled attempts to change how government works to realize a MHiAP approach – although their actual impact and success in driving structural change and substantively integrated practice at scale, remains to be seen. Such efforts challenge not only non-health sectors, but also health and mental health authorities and stakeholders to re-configure their own roles and skills to bridge with others.

In terms of integrated *delivery*, as described above. A range of proven, cost-effective, interventions addressing factors that underlie physical, cognitive, social, and emotional aspects of child development, such as early stimulation, treatment of maternal depression, parent–child dyad interventions and pro-attachment coaching, prevention of early life exposure to violence, abuse, and chronic adversities, and school mental health programs, all have positive mental health outcomes in childhood and later life and can be delivered across multiple settings and sectors. Early childhood development interventions, such as those promoting early attachment, stimulation, and adequate nutrition, together with learning, and appropriate ante-natal and post-natal care (including maternal depression care), are particularly beneficial for disadvantaged children, including children with or at risk for developmental disorders and other mental disorders (Caron *et al*. 2016).

Too often these programs compete over resources, rather than blend them. However, similar core skills and tasks underlie many of these evidence-based programs, which also have overlapping goals (Rotheram-Borus *et al*. 2012). For example, there are several evidence-based programs promoting effective parenting through coaching and feedback to parents in their interactions with their babies and young children. While these approaches may be derived from different theoretical backgrounds, they share common features and skills – skills such as assessment of mother–infant pair interactions, troubleshooting adherence or access, or following parent or child indices – that cut across parenting interventions, and similarly cut across other child and maternal health interventions. Thus, a wide range of interventions we conceptually want to integrate are indeed amenable to integrated delivery through the same personnel, allowing flexible dissemination of a range of synergistic methods within a *shared* workforce.

A growing area of research that supports these kinds of opportunities lies in ‘task-shifting’, or breaking down interventions into core elements that allow them to be more readily assimilated by non-specialist providers or incorporated into the work of professionals across different fields including teachers, or social service and community health workers. Many emerging individual and group therapeutic interventions for children and adolescents in LMICs can be supported through these task-shifted or task-shared models (Kieling *et al*. 2011; Barry *et al*. 2013; Betancourt *et al*. 2013; Reichow *et al*. 2013). While there is a growing evidence base for these building block interventions, more research is needed on their effectiveness and implementation in LMIC settings.

These task-sharing arrangements building off identifying key building block shifts and their distribution, are especially fertile ground for operationalizing and scaling more integrated systems and synergies across fields and platforms (Belkin 2011). The World Health Organization's Mental Health Gap Action Programme Intervention Guide provides evidence-based guidelines for managing priority child mental health conditions in the community and primary-care settings (World Health Organization, 2010). A WHO Parent Skills Training program for caregivers of children with developmental delays and developmental disorders that can be delivered by teams of non-specialist providers is also available for pilot testing.

Examples in LMICs increasingly show that it is indeed possible and feasible to bring together these elements of *policy*, *governance*, and *delivery*. A place- or population-based approach within given catchment areas can facilitate that. An example is the Khayelitsha Child and Adolescent Mental Health Forum, near Cape Town, South Africa. Initiated by a local child psychiatrist, the forum set about mapping all the formal and informal child and adolescent services and resources within the sub-district of this deprived community, and created a regular meeting place for the coordination of a range of different actors. This has enabled the pooling of apparently scarce resources in a manner that has galvanized local providers and established new possibilities for integrated prevention and treatment.

Understanding integration at policy, governance, and delivery levels is important to do at the same time. Supported parenting again illustrates this point. It not only reflects the need for policy to promote the integration of platforms for delivering certain interventions, but echoes the importance of integration in other areas of policy and governance as well. While successful parenting is advanced by these individual ‘hands-on’ methods, it is also advanced through efforts such as income, health, and employment assistance.

#### Improvement

If integration captures different opportunities for the bridging potential of mental health, ‘improvement’ captures the practice of iteratively advancing the effectiveness, innovation, and impact of those efforts. Other integration-driven global health initiatives have lessons for how to approach the complexity and scale such initiatives require. Agreement on quantitative targets and consensus on core best practices appear central to success. Benchmarks for success, and shared methodologies to capture and compare strategies to meet them, however, benefit from specific ‘structures for improvement’.

By structures for improvement, we mean processes for reviewing, reporting, and sharing information that feed back to implementers to create iterative tests for meeting goals in order to improve performance. Setting shared numerical targets for HIV antiretroviral coverage, for example, joined with clear processes for setting consensus over best practices and the collaborative networks and shared methods for reporting results to focus work and compare successful ways to use them, were key elements of success in expanding the use of these medications.

Several initiatives to achieve health Millennium Development Goals incorporated these features, including use of formal, and proven, quality improvement (QI) methods and implementation science tools. Regional QI projects for improved HIV, perinatal, and early child health have shown the value of these methods for system transformation, and with the associated use of more real-time tracking of process and outcome indicators (Barker *et al*. 2007; Franco & Marquez, 2011; Twum-Danso *et al*. 2012; Colbourn *et al*. 2013). The Billion Minds and Lives Early Adopter Network was an early test of the concept that scaled application of QI tools can accelerate integrated models of mental health care in LMICs (www.abillionminds.org). Broader adoption of these methods for improvement and monitoring is especially needed, and well suited, to implementing an integrated child development agenda across diverse cultural groups and in settings with diverse workforce capabilities and system resources.

As mentioned, the international level of consensus meetings and exchanges necessary to establish shared child development aims and benchmarks is increasingly possible in light of the SDGs – but will need focused work to extend that possibility into action. For example, a benchmarked measure of child development that includes attention to socio-emotional development–the Early Child Development Index (ECDI) – has had scaled use and support through UNICEF, and was put forward as an SDG measure (Janus & Offord, 2007). However, better measures are needed. Actionable population measures for early emotional development and for the broadened agenda that comes from converging with a child mental health perspective will need a new generation of tools and commitment to extend their reach.

Similarly, efforts underway in related areas should be opportunities to better craft measuring approaches. For example, WHO, UNICEF, and other partners recently undertook collaborative efforts to define a core set of indicators to monitor progress in implementing the Global Strategy for Women's, Children's and Adolescents’ Health. These include and underscore SDG indicators and goals on promoting mental health and well-being, reducing suicides, and imply that future work should capture rates of depression (WHO, 2016); but like the overall Global Strategy itself, which makes glancing mention of goals for the ‘mental health’ of mothers and their children, including addressing maternal depression (Kuruvilla *et al*. 2016), the effort to measure the success of the Strategy offers an opening, but not yet a secure foothold, to build on; and in the development and tracking of national, regional, and global indicators for SDG targets 3.4 and 3.5, it will be important to modify and expand the opening they provide including to disaggregate indicators by age (at minimum, across early childhood, middle childhood, and adolescence).

We need to move from rhetorical acknowledgment of mental health, to action on child mental health through a model that fully integrates its importance metrically and operationally in a more robust construct of ‘child development’. This is possible to do through some of the already existing major global alliances and driving institutional commitments in global health today; but it will need to purposefully and explicitly build on those efforts, such as the Global Strategy; and that work can be accelerated through the support and wider local use of integrated QI methods.

#### Investment

It will also be accelerated by convergent investment. Bridged strategies and their execution are more likely when there are pooled and converged resources. Given limited investments in these areas separately, convergence could also perhaps better secure and leverage needed levels of investment.

Currently there is significant underinvestment in mental health in general and in child mental health in particular. WHO Mental Health Atlas figures (2011) illustrate how lower income countries spend only 0.5% of their already small health budgets on mental health, whereas high-income countries spend a median of 5.1% of their health budgets on mental health (still a small proportion given the contribution of mental health in overall health). In relation to children, although spending on child and adolescent mental health care is difficult to disaggregate, indications from the WHO Child Atlas (2005) indicate that financing is chiefly from vulnerable sources, such as families’ own out-of-pocket payments, which made up 71.4% of expenditure in African countries, compared with 12.5% in European countries.

The limited matching of resources against the scope and impact of the challenges to children's development that we seek to address is compounded by forces that exacerbate ‘silos’: competition for funding among different disciplines, government agencies, non-governmental organizations, and program categories seeking to defend existing funding streams; but this only in part explains the limited impact organizations at the international and country level (UNICEF, Save the Children, CARE, others) have had in impacting or leveraging child mental health. Aligning resources more successfully will need more than attitude change, but entirely new structures and scale of funding.

Other global strategies offer lessons in building the kind of architecture for action needed for a child development agenda in general, and especially for an agenda that highlights the bridging role of capabilities to improve and protect child mental health. A ‘Global Fund’ for child mental health and development, for example, could mobilize action that operationalizes innovation as well as broader participation in investing in child mental health.

While overall the SDGs missed opportunities to more specifically detail the salient connections between child development and social development, its inclusion in the SGDs, and the broader understanding of human capital as a driver of sustainable development that the SDGs advance, offers real opportunity to define a comprehensive child development agenda as a discrete area of investment. Such resources should be earmarked for workforce and infrastructure to implement the kinds of integrated approaches advocated for here.

## Recommendations for action – GAC-MHiAP

We therefore call on prominent conveners of child development and survival coordinated action, in particular the United Nations as part of its launching of the SDGs, the Child Survival Call to Action Network as part of its Global Roadmap Initiative, and the World Bank, especially in the context of the recent highlighting of mental health and development at its 2016 Spring Meetings, to convene a broadly representative Planning Summit for Child Development and Mental Health.

The charge of the Summit would be to agree upon a set of goals and organizational planning steps for a new Global Alliance for Child Development and Mental Health in All Policies (GAC-MHiAP). Such an Alliance should be charged to facilitate connections between action on child mental health and the SDGs through concerted attention on integration, improvement, and investment. These three areas as detailed in this white paper offer a framework for the formation of an initial working group and report-out recommendations for this Summit and for the work of GAC-MHiAP moving forward [Table 1].

As with progress in the delivery of child survival-related interventions, the maturing of key global networks and institutions provide the foundation for the long overdue inclusion of child mental health aims, measures, and expertise in global child health strategies. Key, established child survival and development actors such as the Action Network, and the Global Financing Facility of the World Bank are readily positioned to successfully co-lead the Summit and Alliance. Such anchor conveners and partners are especially important to bring together the broad array of institutions, sectors, experts, and advocates necessary for global convergence on a complete vision of healthy child development and mental health – from economists and educators, to gender equity advocates and government reformers. Success in that vision means actually, not just conceptually, moving beyond the ‘usual suspect’ leaders in child development and mental health.

It is long overdue for the global health community to take on the question of how to sponsor global governance, national and regional policy, and accessible local delivery that advance a comprehensive approach to child development. Science, momentum, and population needs, all point to the possibility and imperative to do so through leveraging platforms for maternal and child health, and their alignment with social development efforts and outcomes more broadly.
